# Endoscopic Management of Difficult Biliary Stones: An Evergreen Issue

**DOI:** 10.3390/medicina60020340

**Published:** 2024-02-19

**Authors:** Magdalini Manti, Jimil Shah, Apostolis Papaefthymiou, Antonio Facciorusso, Daryl Ramai, Georgios Tziatzios, Vasilios Papadopoulos, Konstantina Paraskeva, Ioannis S. Papanikolaou, Konstantinos Triantafyllou, Marianna Arvanitakis, Livia Archibugi, Giuseppe Vanella, Marcus Hollenbach, Paraskevas Gkolfakis

**Affiliations:** 1 Department of Gastroenterology, “Konstantopoulio-Patision” General Hospital of Nea Ionia, 14233 Athens, Greece; mantmagda@gmail.com (M.M.); g_tziatzios@yahoo.gr (G.T.); konparaskeva@gmail.com (K.P.); 2 Department of Gastroenterology, Post Graduate Institute of Medical Education and Research, Chandigarh 160012, India; shahjimil22@gmail.com; 3 Endoscopy Unit, Cleveland Clinic London, London SW1X 7HY, UK; appapaef@hotmail.com; 4 Department of Gastroenterology, General University Hospital of Larissa, 41110 Larissa, Greece; vaspapadopoulos82@gmail.com; 5 Gastroenterology Unit, Department of Medical Sciences, University of Foggia, 00161 Foggia, Italy; antonio.facciorusso@virgilio.it; 6 Gastroenterology and Hepatology, University of Utah Health, Salt Lake City, UT 84132, USA; daryl.ramai@hsc.utah.edu; 7 Hepatogastroenterology Unit, Second Department of Internal Medicine—Propaedeutic, Medical School, National and Kapodistrian University of Athens, Attikon University General Hospital, 12462 Athens, Greece; ispapn@hotmail.com (I.S.P.); ktriant@med.uoa.gr (K.T.); 8 Department of Gastroenterology, Hepatopancreatology, and Digestive Oncology, University Hospital of Brussels (HUB), 1070 Brussels, Belgium; marianna.arvanitaki@hubruxelles.be; 9 Pancreato-Biliary Endoscopy and Endosonography Division, Vita-Salute San Raffaele University, 20132 Milan, Italy; livia.archibugi@hotmail.it (L.A.); g.e.vanella@gmail.com (G.V.); 10 Medical Department II, Division of Gastroenterology, University of Leipzig Medical Center, D-04103 Leipzig, Germany; marcus.hollenbach@web.de

**Keywords:** choledocholithiasis, ERCP, sphincteroplasty, mechanical lithotripsy, cholangioscopy

## Abstract

Choledocholithiasis is one of the most common indications for endoscopic retrograde cholangiopancreatography (ERCP) in daily practice. Although the majority of stones are small and can be easily removed in a single endoscopy session, approximately 10–15% of patients have complex biliary stones, requiring additional procedures for an optimum clinical outcome. A plethora of endoscopic methods is available for the removal of difficult biliary stones, including papillary large balloon dilation, mechanical lithotripsy, and electrohydraulic and laser lithotripsy. In-depth knowledge of these techniques and the emerging literature on them is required to yield the most optimal therapeutic effects. This narrative review aims to describe the definition of difficult bile duct stones based on certain characteristics and streamline their endoscopic retrieval using various modalities to achieve higher clearance rates.

## 1. Introduction

Gallstones are one of the most common findings during abdominal ultrasounds, with a prevalence of 6% in men and 9% in women, regardless of the presence of symptoms [1]. Among these patients, common bile duct (CBD) stones are found in 1–15% [2]. Bile stones can be formed either in the gallbladder or de novo in the CBD, and most of the time they consist of cholesterol; calcium bilirubinate stones are the second most frequent type of gallstones. Due to the high risk of serious complications (obstructive jaundice, cholangitis, pancreatitis) even in patients with asymptomatic CBD stones, it has been recommended by the European guidelines that CBD stones should be extracted in all eligible patients who are fit to undergo an intervention [3]. Endoscopic retrograde cholangiopancreatography (ERCP) is a commonly used method for CBD stone removal according to current guidelines by various societies [3,4,5]. Biliary sphincterotomy and stone extraction with balloon or basket catheters are usually efficient in treating the vast majority of CBD stones; still, these conventional techniques are unsuccessful in 10–15% of cases where difficult bile duct stones are detected [6].

The aim of this review is to summarize and present the existing evidence on the characteristics of difficult stones and the existing modalities to manage them.

## 2. Definition of Difficult Bile Stones

**Figure 1:**
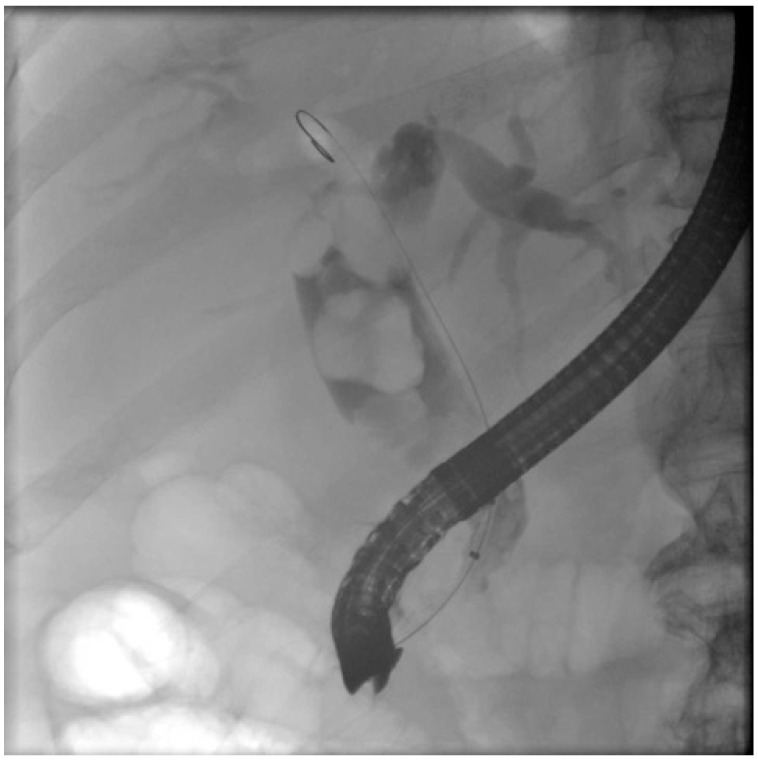
Fluoroscopy image demonstrating multiple large common bile duct stones.

Although a plethora of factors has been associated with the failure of standard ERCP methods, there are no certain correlations. Thus, the term difficult biliary stone describes any stone found in the bile duct (common or hepatic) which cannot be removed by a single conventional ERCP session, including biliary sphincterotomy and stone extraction with balloon or basket catheters [7]. This may be attributed to specific stone characteristics demonstrated during magnetic resonance cholangiopancreatography (MRCP) (such as size, number, shape, consistency, and location of the stone) or certain biliary anatomy (biliary variants) or patient traits (comorbidities), as discussed below:

Stone characteristics: One of the most important characteristics defining a difficult stone is its size. In general, a stone diameter >1.5 cm is associated with difficulty in endoscopic extraction (Figure 1). In one prospective study, a stone diameter of 2.2 cm was proposed as a cutoff predictor for multiple ERCP sessions [8]. Apart from the large size, the detection of multiple stones also decreases the ERCP success rates [9]. However, a retrospective study demonstrated that the number of stones was less important compared to the size, in terms of ERCP effectiveness [9]. Another stone attribute is the shape; for instance, peculiar or barrel-shaped stones tend to be retrieved more arduously [7]. Similarly, both the hard consistency and the location of the stones are contributing features of strenuous ERCPs. For example, intrahepatic or cystic duct stones, or those that are located above a stricture, or are impacted are considered as difficult [3,5,10]. 

Bile duct anatomy: Biliary anatomy is one of the important factors to consider in defining a difficult biliary stone. Bile duct size below the stone is particularly important in determining the difficulty of passing the stone through during the extraction attempt. The presence of an oblique, narrowed, sigmoid-shaped distal biliary duct, periampullary diverticula, or an acute angulation (≤135°) of distal CBD are important factors to consider when defining a difficult biliary stone [10]. Similarly, the presence of strictures under the stone (stone–stricture complex)—due to inflammation, iatrogenic complication, or sclerosing cholangitis—also makes endoscopic retrieval challenging [11,12].

Patient-centered care: The patients’ comorbidities, including advanced age, cardiopathies, and impaired coagulation may play a crucial role during an ERCP procedure and decision making [13]. According to the recent European Society of Gastrointestinal Endoscopy (ESGE) guidelines, P2Y12 receptor antagonist antiplatelet agents should be suspended 7 days prior to the ERCP in case of sphincterotomy, while in high-risk patients with coronary artery stents, personalized assessment strategies should be implemented, along with cardiology review. Warfarin and direct oral anticoagulants should be discontinued 5 and 3 days accordingly prior to the procedure. In the meantime, bridging therapy with low molecular weight heparin should be administered to patients with high thrombotic risk, such as prosthetic metal heart valves. Aspirin should be suspended prior to ERCP only when papillectomy is planned. Regarding their restart, P2Y12 receptor antagonists are re-initiated 1 to 2 days after the procedure, warfarin and low molecular weight heparin directly post-ERCP until reaching the proper INR levels (when heparin is discontinued), and direct oral anticoagulants 2 to 3 days after the ERCP [14].

Despite that, there is heterogenicity in the definitions of difficult biliary stones across various guidelines; those larger than 1–1.5 cm, or with unusual shape (barrel-shaped or eccentric stone), or in atypical locations (intrahepatic or inside the cystic duct), impacted stones with an abnormal distal duct (oblique, narrowed, peri-diverticular), the presence of a narrow or sigmoid-shaped bile-duct and short length or angulation <135° of the distal CBD, or altered anatomy such as Billroth-II or Roux-en-Y anastomoses or previously failed endoscopic attempt for stone extraction, are defined as difficult biliary stone [3,4] (Table 1).

## 3. Endoscopic Management of Difficult Biliary Stones

### 3.1. Optimization of Conventional ERCP Techniques

Some of the commonest reasons for failed endoscopic attempts of biliary stone removal are inadequate endoscopic sphincterotomy (EST), inappropriate use of extraction devices (balloon and basket catheters), and suboptimal endoscopic papillary balloon dilation (EPBD). EST is the initial step in cases of bile duct stone extraction. The technique was initially described in the literature by Kawai et al. [15] and by Classen and Demling in the early 1970s, and involves the selective cannulation of the CBD and the transpapillary insertion of a knife (sphincterotome), through which a high-frequency current is applied in order to cut the biliary sphincter [16]. In a randomized control trial (RCT), published by Karsenti et al., the CBD clearance rate was 74% among 73 patients with bile stones ≥13 mm, when EST was solely used [17]. Although success rates of up to 90% have been reported in older studies, the percentages also declined in the presence of large bile stones (>15 mm) [18]. Regarding the extent of sphincterotomy, limited sphincterotomy, recommended by ESGE guidelines, implies that it is performed up to the transverse hood, while complete sphincterotomy extends up to the superior margin of the intramural bile duct. So far there is no head-to-head comparison regarding the stone extraction rate of these two techniques; still, limited sphincterotomy is proposed in the guidelines as it has been associated with a lower risk of bleeding [3]. 

Instead of sphincterotomy, endoscopic papillary balloon dilatation (EPBD)—first reported in the 1980s [19]—involves the insertion of a small balloon (≤10 mm) into the biliary orifice. The proposed balloon diameter by ESGE guidelines is 8 mm in EPBD, irrespective of the CBD diameter. Additionally, ESGE suggests that EPBD duration, following waist disappearance, should exceed 2 min to reduce the risk of PEP [20]. A prospective study of 170 patients that compared 1-min versus 5-min EPBD regarding long term outcomes in a 7-year follow-up period, including recurrent episodes of choledocholithiasis or acute cholangitis, exhibited no clinically relevant differences [21]. On the other hand, in 2020 researchers displayed that when EPBD duration was less than 3 min (compared to over 5 min), the risk of PEP was higher (*p* = 0.032) [22]. RCTs comparing EPBD to EST had controversial results. While in a German study EPBD exhibited lower stone removal rates during the index ERCP attempt compared to EST, 2 years later in a Japanese study the overall success rates were similar in the two groups [19]. A meta-analysis that compared EPBD to EST about bile stone management revealed that although the initial success rates were higher in the EST group (79% vs. 70%), the overall stone extraction rates were similar. Still, in 20% of cases in the EPBD group, stone extraction was incomplete; hence, “rescue” EST was performed with or without the aid of mechanical lithotripsy (ML) [23,24]. The same results were demonstrated in another meta-analysis by Liu et al. with statistically insignificant differences regarding stone removal between the two groups (95% in the EPBD group versus 96% in the EST group; *p* = 0.36). Additionally, ML was required more frequently in the EPBD group (*p* = 0.0004) [25]. Conversely, Weinberg et al. showed that EPBD was less efficient during both the first and subsequent ERCP sessions in terms of stone removal [26]. 

In a recent study, a combined ERCP approach using EST plus EPBD manifested higher stone removal rates when compared to EST alone. In this approach, the complication rate was also lower and both the procedure time and the need for mechanical lithotripsy were diminished [27]. 

**Figure 2:**
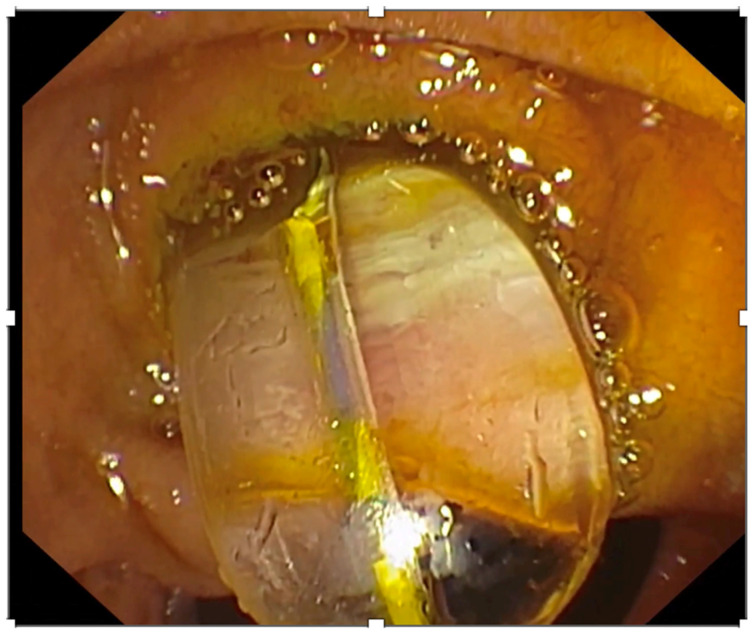
Endoscopic image of a balloon used to perform endoscopic papillary large balloon dilatation.

Endoscopic papillary large balloon dilatation (EPLBD), developed a few years after EPBD in the early 2000s [19], provides an option to enhance the complete stone extraction percentage following EST [28]. PEP percentages after EPLBD tend to be lower compared to EPBD, a feature probably attributed to the ample papillary dilatation [29]. Additionally, limited EST that precedes EPLBD is proposed in order to control the direction of luminal tearing when dilation occurs [3,30]. Nowadays, limited sphincterotomy followed by endoscopic papillary large balloon dilation is considered the first-line approach in terms of difficult CBD stones, according to ESGE guidelines (Figure 2). Similarly, the American Society for Gastrointestinal Endoscopy (ASGE) guidelines suggest performing endoscopic sphincterotomy followed by large balloon dilation in cases of large choledocholithiasis (≥1 cm) [5]. This suggestion was based on nine randomized control trials (RCTs) where the maximum size of papillary balloon dilation was 20 mm. A retrospective study by Kuo et al. highlighted the superiority of limited EST combined with EPLBD in stone removal when compared to complete EST and EPLBD alone, in patients with large bile duct stones (>15 mm). In detail, stone extraction rates during the first ERCP session were higher in the combined therapeutic group (*p* = 0.032), while the need of ML was lower [31]. In randomized controlled trials when EST was compared to EPLBD, head-to-head data from 19 Japanese centers in cases of large bile stones (≥10 mm) revealed that single-session complete stone removal percentages were higher in the EPLBD group than in the EST (90.7% versus 78.8%; *p* = 0.04). In this study, adverse effects did not show significant difference [32]. In another trial, EST plus EPLBD and EPLBD alone demonstrated similar stone extraction and adverse events rates, with the EPLBD alone group taking a longer time during the session (*p* = 0.08) [33]. An RCT that studied all those types (EST, EPLBD, and their combination) in patients with choledocholithiasis concluded that their success and adverse effects rates were comparable [34]. 

There are only a few meta-analyses comparing the efficacy of EPLBD alone in terms of stone extraction rates with a combined technique EST/EPLBD. A systematic review published in 2019 compared 369 patients who underwent EPLBD alone with 367 patients who underwent EPLBD following EST for stone removal. Researchers concluded that both options were equally effective in the initial endoscopy [35]. Similarly, adverse effects and procedure time showed no differences. Based on the JGES, complete stone clearance at the first session of EST fluctuated between 56.2% and 92.7% using the conventional methods [36], while in the case of EPLBD the percentages were higher and varies between 80.9% and 89% for the initial treatment and between 95.2% and 100% after any extra ERCP session [28]. A network meta-analysis that compared EST with EPBD and their combination (EST+EPBD) among 3726 patients with CBD stones concluded that the combined therapy yielded a higher stone extraction rate in the first ERCP session when compared to EPBD or EST, but this result was statistically non-significant. Nevertheless, EST success rates were higher that EPBD in terms of stone removal [37]. Regarding the duration of an inflation of the balloon, a recent multi-centric RCT has shown that balloon inflation for 0/30/60/180/300 s had equal efficacy in stone extraction; however, rates of post-ERCP pancreatitis were higher in 0 or 300 s of inflations. The authors concluded that a balloon dilation time of 30 s is optimum for clinical success and to reduce frequency of post-ERC pancreatitis [38]. Generally, EST and EPLBD are the first steps during the ERCP and they are highly efficient when performed by experienced endoscopists. 

#### Extraction Devices

Regarding stone retrieval, there are two different types of extraction device which can be used after EST, EPBD or EPLBD: extraction balloon catheters and basket catheters. Biliary extraction balloon catheters vary in size between 6 and 20 mm [39], while extraction baskets are commercially available in a plethora of types too, such as angular fold, ball, and spiral type [40]. 

Both extraction devices are considered equally effective according to ESGE guidelines [3]. However, in a recent multicenter retrospective study with 904 patients who underwent ERCP for choledocholithiasis, balloon catheters exhibited significantly higher success rates for stone extraction during the first ERCP session when compared to basket catheters (81.3% vs. 17.8%; *p* < 0.001) [41]. Still, this study included only patients with small CBD stones ≤10 mm and its results should be re-evaluated in cases with difficult bile duct stones.

Similarly, a study published in 2022 demonstrated higher success rates with balloon catheters compared to basket catheters regarding ≤10 mm stone extraction [42]. Nevertheless, retrieval baskets are evolving and in 2023 an experimental study analyzed seven different types of basket catheters according to their radial and axial force measurements, in order to review their mechanical properties and promote basket development in the future [43]. Inoue et al. displayed that helical eight-wire baskets with smaller interwire spaces at the tip are fundamental when extracting corner pocket stones, and the addition of rotation enhances the overall success [43]. 

### 3.2. Mechanical Lithotripsy (ML)

**Figure 3:**
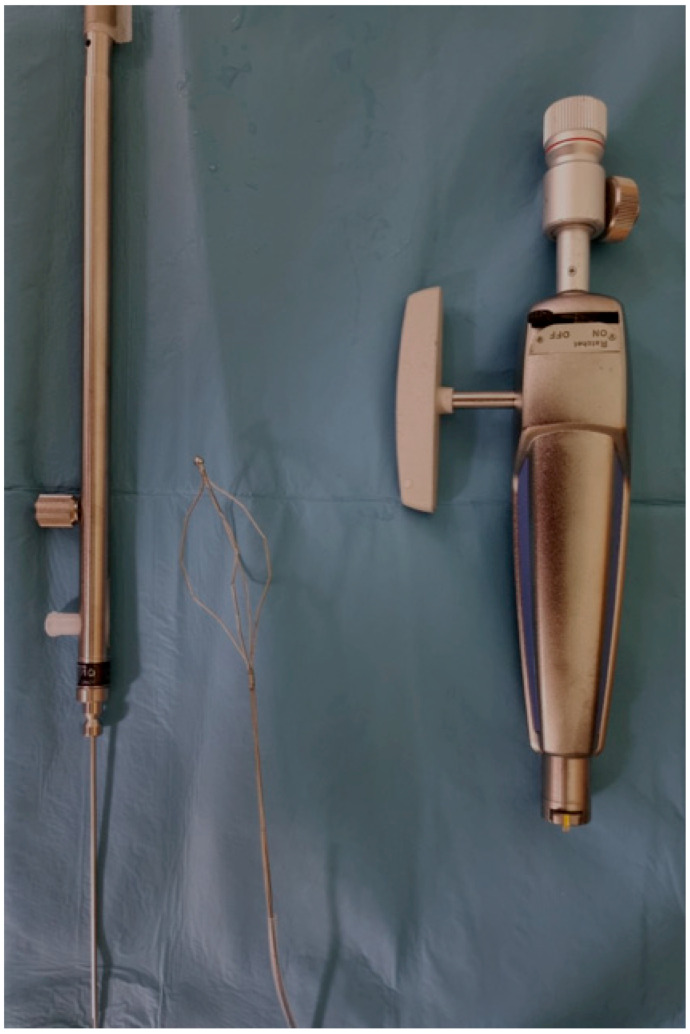
One of the available sets to perform mechanical lithotripsy.

The wide use of EST and EPLBD as a primary treatment during ERCP led to a 30–50% reduction of additional stone retrieval method usage, such as mechanical lithotripsy (ML) [44]. Still, ML is used in several cases to achieve complete stone extraction, with cost-effectiveness being its main advantage over alternatives [45]. There are two types of ML catheter: integrated and salvage devices. Integrated devices consist of a wire basket, a metal sheath, and a handle, all used through the operating channel (Figure 3). They can be used as a standard basket until lithotripsy is required. Meanwhile, salvage devices lack the wire basket since they are mostly used in the occurrence of stone or basket impaction [45]. Up to 80–90% success rates have been reported by ML as a salvage therapy, while it seems to be quite effective even in large bile duct stones (68% in stone clearance over 28 mm) [45]. In a retrospective study, bile duct stones >15 mm were extracted in 272 out of 304 patients by ML after the failure of EST with a 90% success rate. Complete removal during the initial ERCP was achieved in 211 patients, while the rest underwent multiple lithotripsy sessions [46]. In 2023, a retrospective study analyzed the treatment outcomes of EPLBD alone when compared to the combination of EPLBD and ML in terms of recurrent choledocholithiasis, during a 4-year follow-up period. Researchers showed that the long-term recurrence rate was higher in the combination group, but stone size did not exceed 15 mm in all cases, which is a restrictive parameter of the final outcome [47].

The most common adverse effects during ML are basket and stone impaction, which are reported in up to 6% of cases, regardless of the stone diameter [48]. When ML was compared to EPLBD in cirrhotic patients with CBD stones, the stone clearance rate was slightly inferior without reaching statistical significance (93.8% vs. 98%), but the adverse events were higher (*p* = 0.04). Consequently, the latest European guidelines suggest the use of ML only after the failure of EST and EPLBD [3]. Thus, ML is commonly reserved for more complicated cases. 

### 3.3. Extracorporeal Shock Wave Lithotripsy

An alternative method is extracorporeal shock wave lithotripsy (ESWL), which can be performed when mechanical lithotripsy is unsuccessful [3,49]. During this procedure, high-pressure electrohydraulic or electromagnetic energy is delivered extracorporeally, aiming to fragment the stones. This is performed under fluoroscopic guidance, via a naso-biliary drain, which targets the bile duct stones. Tao et al. used this technique in patients with biliary stones after a failed initial ERCP attempt. In detail, researchers compared ESWL followed by ERCP versus ERCP alone, which resulted in a higher total ductal clearance in the first group (*p* = 0.029), as well as higher clearance of stones >30 mm (*p* = 0.016). Procedure time was also significantly shortened with the use of ESWL (*p* = 0.034) [50]. However, it is also associated with a plethora of adverse effects, such as hematomas, cardiac arrhythmias, biliary obstruction, and hemobilia [45]. ESWL’s availability is limited, while its safety and efficacy are controversial since it often requires the placement of a naso-biliary catheter for optimum opacification of the stone with additional procedures for extracting the remaining stone fragments and inevitably elevating the financial burden; therefore, it is rarely used in daily practice and should be reserved for cases of failure of the other conventional techniques or when cholangioscopy is unavailable [3].

### 3.4. Cholangioscopy Guided Lithotripsy

**Figure 4:**
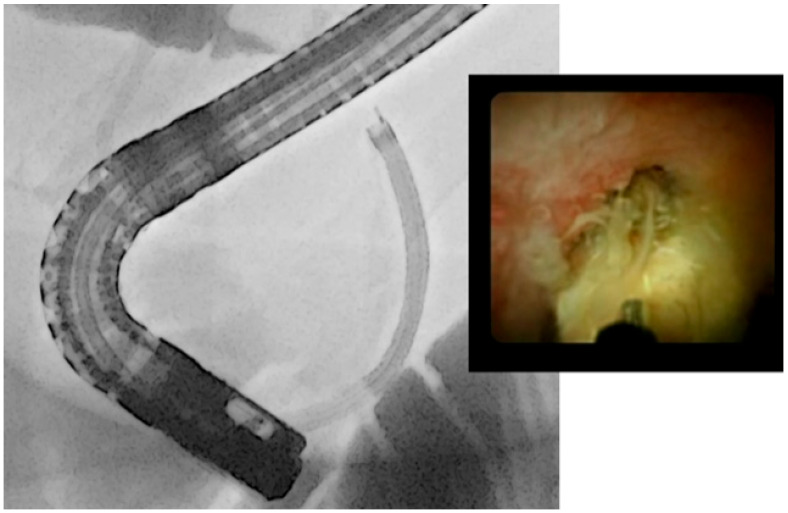
Endoscopic and fluoroscopic image of single-operator cholangioscopy used to treat a difficult biliary stone.

Advanced ERCP procedures have also emerged, such as single operator cholangioscopy (SOC) with holmium laser lithotripsy (LL) or electrohydraulic lithotripsy (EHL) [7]. Cholangioscopy was first introduced in the early 1970s as a dual-operator cholangioscopy, also known as “mother–daughter cholangioscopy” (MDC) [51]. This cumbersome technique was soon replaced by single-operator cholangioscopy (SOC) with a fiber scope in its first generation (SpyGlass; Boston Scientific, Marlborough, MA, USA) while its second generation introduced a digital scope around the mid-2000s (SpyGlass DS, D-SOC) [52]. Apparently, the D-SOC system improves the diagnostic yield during ERCP for pancreaticobiliary diseases and it also diminishes the procedure time, resulting in minimum radiation exposure, compared to fiber scopes SOC [53] (Figure 4). Direct peroral cholangioscopy (DPOC) is a technological advancement that has also been introduced to the market with a larger working channel and electronic chromoendoscopy capabilities [54]. However, DPOC appears more challenging since there is a risk of gastric looping, while cases of air embolization have also been reported. In 2019, an observational study was published with 79 patients undergoing DPOC after an initial ERCP procedure with incomplete stone extraction. The ultra-slim scope was inserted immediately into the CBD of only 14 patients, while a guide wire and overtube assistance were used in 54 and seven patients respectively to facilitate the procedure. DPOC was unsuccessful in four patients [55].

Laser lithotripsy (SOC-LL) and electrohydraulic lithotripsy (SOC-EHL) are additional methods employed to fragment difficult stones that cannot be removed by conventional techniques during ERCP. Laser lithotripsy (SOC-LL) in the context of ERCP began to gain traction in the late 1980s and 1990s. It generates a high-energy pulsed shock wave with minimum thermal injury to the nearby biliary epithelium due to its rapid (within fractions of a second) function, ultimately leading to stone fragmentation [56]. Two types of LL are currently used with similar results: holmium—yttrium aluminum garnet (YAG) laser, and frequency-doubled double-pulse neodymium—YAG laser (FREDDY) [57].

Electrohydraulic lithotripsy (SOC-EHL) is the second device with a different mechanism of action that has been used since the early 1980s for the fragmentation of bile duct stones [58]. Its functional principle is based on the high electric voltage between two isolated electrodes which are placed at the tip of a fiber. Thus, electric sparks are created which in turn provoke an immediate expansion of the surrounding liquid, leading to a spherical shock wave and finally to stone fragmentation [59]. In order to be used, the rigid EHL fiber is advanced through the accessory channel of the D-SOC, but caution must be taken to avoid any damage to the flexible endoscope. Hence, prior to the EHL insertion, straightening of the endoscope is recommended [60]. 

According to ESGE guidelines, cholangioscopy-assisted intraluminal lithotripsy techniques (electrohydraulic or laser) are equally safe and effective in cases of difficult bile duct stones, after the conventional first-line approach [3]. However, ASGE guidelines remain neutral between intraductal therapy (electrohydraulic or laser lithotripsy) and papillary dilatation, when difficult choledocholithiasis is suspected due to large stone size (≥1 cm) or anatomic considerations such as stone impaction, proposing that local expertise, cost, and individualized preferences should be considered [5]. This dead end was mainly attributed to the presence of only one RCT and the lack of enough data to support any unambiguous suggestions. Still, due to its effectiveness, cholangioscopy should be considered an integral part in difficult bile stone management. 

Advanced cholangioscopy techniques have been widely available for over a decade; still, there are only a few associated RCTs. In 2018, American researchers published an RCT comparing D-SOC-LL to conventional techniques including ML and EPLBD in patients with large bile stones (>1 cm) [61]. Sixty cases were studied in total. Duct clearance rates in the D-SOC-LL group were higher (93%) than the conventional group (67%; *p* = 0.009), but at the same time their procedure duration was longer (120.7 ± 40.2 min versus 81.2 ± 49.3 min, *p* = 0.0008). Two years later, another RCT compared the same variables, recruiting an equally small number of patients (66 in total). Likewise, stone clearance rates were higher in the D-SOC-LL group (93.9%) versus the conventional group (72.7%; *p* = 0.021), but in this publication additional parameters were studied, such as stone size and bile duct anatomy. It was shown that stone/duct ratio ≤ 1 and the lack of a tapered bile duct were associated with a better outcome, defined as stone clearance [62]. A RCT from Thailand endeavored to highlight the superiority of D-SOC-LL against ML in adults that had already undergone EST and EPLBD in a previous ERCP, due to cholelithiasis. The initial failure of conventional methods could be attributed to the presence of “difficult” bile stones. This RCT, comprising of 32 patients who were equally distributed to the two groups, demonstrated that D-SOC-LL had 100% efficacy (16/16 patients) in stone extraction during the first session, compared to ML with 63% efficacy (10/16 patients; *p* < 0.01). In the second group, three patients who were immediately treated with D-SOC-LL after the failure of ML also had a favorable result with complete stone removal [63], underlying the potency of D-SOC, despite the small number of participants. Favorable results for D-SOC were also demonstrated by Turowski et al. with 91.1% removal of bile duct stones among 107 patients, while at the same time 75 patients received EHL (71 of which were successful) [64]. D-SOC efficacy was shown in a multicenter, prospective study by Fugazza et al. too, with 92.1% complete duct clearance percentage, 82.1% of which were achieved during the first session [65]. 

A meta-analysis with 1762 participants published by McCarty et al. compared LL and EHL in terms of overall and single-session stone fragmentation success rates. Although the overall success rates did not differ between the two groups (90.14% in EHL compared to 92.90% in LL), the single-session lithotripsy success rates were statistically significantly lower in the EHL group compared to LL (70.85% versus 82.97%; *p* = 0.021). It was also demonstrated that cholangioscopy along with LL had statistically significant shorter procedure times compared to EHL and no difference in adverse effects [66]. Similar results to the latter conclusion were described in an international multicenter retrospective study in 2018 [67]. SOC-LL was compared to SOC-EHL in 407 patients with difficult biliary stones and the mean procedure time in the SOC-LL group was clinically significantly reduced (49.9 min compared to 73.9 min in SOC-EHL, *p* < 0.001). Still, the comparison of duct clearance rates between those groups revealed no clinically meaningful difference (77.4% in the first ERCP procedure and 97.3% overall rate), as did the adverse effects (3.7%).

The most inclusive meta-analysis so far—published in 2023—compared all the available techniques of stone removal during ERCP, with duct clearance being the primary endpoint and the percentage of adverse effects the secondary endpoint [68]. SOC was a superior method of duct clearance, and it was demonstrated that ML was the least effective procedure followed by EST. This study reinforces the utility of SOC, and more endoscopists should be trained to perform this technique. 

Machine learning approaches and artificial intelligence could also contribute to the detection of difficult bile duct stones by standardized scores and classification systems. Huang et al. recently published a study with 173 patients demonstrating that a specialized computer software could successfully identify the existence of strenuous bile stones using cholangiogram images [69]. Thus, cholangioscopy could be introduced early at proper timing in specific cases. 

#### 3.4.1. Cost-Effectiveness 

From a financial point of view, the total cost varies among the endoscopic approaches. According to a randomized clinical trial, the combination of EST and EPLBD was more cost-effective when compared to EST alone, due to the reduced need for ML [70]. 

This was confirmed by Paik et al. as well, but in their study the cost-effectiveness of EST and EPLBD combined was compared to EST followed by ML. They demonstrated that the mean cost per patient was lower in the first group (USD 1644 versus 1225, respectively; *p* = 0.04) [71]. On the other hand, ESWL comes with an inherently increased cost; therefore, it should not be considered as part of the first-line cost-effective approaches [72]. 

In 2021, Canadian researchers published a decision tree model about the proper timing of SOC-EHL in cases of difficult choledocholithiasis. They compared early SOC-EHL performed during the first ERCP (SOC-1) with SOC-EHL during the second and the third ERCP attempt (SOC-2 and SOC-3 respectively), deducing that SOC-1 was the most cost-effective strategy. The authors also underlined that patients’ quality of life assessment is equally important and should be considered, minimizing extra hospital admissions and post-operative risks. Thus, the early detection of difficult biliary stones is mandatory, leading to immediate cholangioscopy, even during the first ERCP [73]. Analogous findings based on decision tree models were displayed by Deprez et al. associated with the treatment of difficult bile duct stones and the diagnosis of bile duct strictures [74], while other authors in 2022 agreed that SOC performed as a second-line therapy would be the ideal approach [75]. Despite SOC’s higher cost when compared to conventional methods, the higher percentages of stone depletion may call for its reappraisal.

#### 3.4.2. Adverse Events

Adverse effects have also been reported during all the previously mentioned modalities, the most prevalent being bleeding, perforation, infection (usually cholangitis), and pancreatitis. Bleeding was mainly associated with complete EST but rates have lately decreased due to the wide use of limited EST [3]. When a site of bleeding is detected, endoscopic hemostasis is usually sufficient. Likewise, in cases of perforation, confirmed by a CT-scan, antibiotics are always needed along with early surgery consultation. However, the ultimate management usually depends on the perforation size; if relatively small, a bile duct drainage and nasogastric tube placement constitute a conservative approach [36]. Antibiotics are also used in cases of cholangitis or pancreatitis. Regarding the latter, ESGE has published guidelines for post-ERCP pancreatitis prophylaxis recommending rectal administration of 100 mg of diclofenac or indomethacin immediately before or after ERCP in all patients without contraindication. Moreover, in high-risk patients, the placement of a 5-Fr prophylactic pancreatic stent is suggested [76]. PEP risk is also paradoxically higher in EPBD than in EPLBD or EST, probably due to insufficient papillary dilation. If ML is subsequently used, the risk of PEP remains high [29]. 

During ERCP for stone extraction, biliary basket impaction has also been reported, with percentages fluctuating between 0.8–5.9%. Most of the time it was treated with ML. Still, cases of basket handle cord break have already been reported in the bibliography, which required the extra use of SOC-LL, after the failure of ML [77,78]. 

As previously reported regarding the conventional techniques, when comparing EPLBD along with the combination of EPLBD/EST the adverse event rates were similar [34,35]. On the other hand, in cirrhotic patients, adverse events after ML were significantly more common when compared to EPLBD, as stated in the European guidelines [3]. In cholangioscopy, post-operational adverse events were similar when D-SOC-EHL was compared to D-SOC-LL [66,67]. 

Generally, the percentage of post-ERCP complications is 7%, regardless of the procedure indication, while in the case of D-SOC rates up to 16.4% have been reported. When the latter is performed due to bile duct stone extraction, the procedure time tends to be longer compared to diagnostic procedures, and consequently the adverse events might be increased [79]. Still, in a retrospective analysis from 22 tertiary centers worldwide, only 3.7% of patients treated with D-SOC due to difficult bile duct stones demonstrated any adverse event [67]. In a meta-analysis that included both cases of difficult bile duct stones and strictures, the adverse event rate was 7%, 4% of which included cholangitis and 2% pancreatitis [80]. Similar results have been reported in several studies, implying that cholangitis is the predominant adverse event post D-SOC due to bile duct stones which may be due to continuous irrigation with copious amounts of fluid during the procedure [79]. Even in this case, peri-interventional antibiotic prophylaxis may decrease the percentage of cholangitis, as was displayed by a multicenter retrospective cohort study [64].

In a systematic review by Galetti et al., D-SOC was compared to conventional ERCP techniques in terms of adverse events. Among three RCTs and 43 observational studies which included patients with complex bile duct stones regarding size (>15 mm or stone size larger than the CBD diameter), stone number (multiple stones), bile duct anatomy (altered postoperative anatomy), and atypical stone location (intrahepatic), no significant difference was detected [81]. However, in centers of expertise, the rate of adverse effect percentages tends to be lower. 

### 3.5. Choledocholithiasis in the Presence of Altered Anatomy

The presence of choledocholithiasis with altered anatomy poses a unique challenge in its endoscopic retrieval. In the presence of altered anatomy following Billroth II surgery, the use of balloon enteroscopy-assisted ERCP is suggested. On the contrary, Billroth I surgery leaves intact the track towards the ampulla of Vater, permitting standard ERCP procedures. However, due to the rise of bariatric surgeries, the Roux-en-Y gastric bypass is the most common anatomical modification, in which either balloon enteroscopy-assisted ERCP or endoscopic ultrasound-directed transgastric ERCP (EDGE procedure) is performed [49,82,83]. The EDGE procedure was first introduced in 2014 [84], and includes the placement of a lumen-apposing metal stent (LAMS) under endoscopic ultrasound which connects the jejunum or gastric pouch to the excluded stomach, in order for the duodenoscope to be promoted. Although promising, reports about the formation of fistulae after stent removal tend to overshadow this technique; still, large-scale data are lacking. James et al. published a report about an EDGE case series with 12 out of 19 patients developing fistulae [85], successfully treated by argon plasma coagulation [85]. Over-the-scope clip placement or revisional surgery have also been proposed for the management of fistulae closure [49]. Other adverse effects of this method include stent migration and bleeding. In 2020, a meta-analysis compared EDGE, balloon enteroscopy-assisted ERCP, and laparoscopic ERCP (Rendezvous technique) with procedure completion as the primary and adverse effects as secondary endpoints [86]. The authors concluded that both EDGE and laparoscopic ERCP had higher success rates than balloon enteroscopy-assisted ERCP, while the latter was the safest. A current published retrospective analysis contradicts those results, showing that the mean number of endoscopic sessions to achieve stone depletion was lower in balloon enteroscopy-assisted ERCP, when compared to EDGE (*p* = 0.01). In the same study, complete stone removal rates were significantly higher in the first group (*p* = 0.009), at the cost of increased procedure time (*p* = 0.001) [87]. 

Endoscopic ultrasound (EUS) guided gastrojejunostomy is another variation of ERCP, similar to EDGE, in altered post-surgical anatomy with the placement of LAMS connecting the gastric stump to the biliary afferent loop [88]. 

Since our review mainly focuses on the endoscopic management of bile duct stones, alternative options including surgical bile duct exploration will not be discussed.

#### Hepatolithiasis

Uncommon bile stone locations are another consideration that pose treatment challenges. Liu et al. described a case of right posterior intrahepatic duct dilation caused by multiple stones. Although hepatectomy tends to be the norm, the researchers managed to remove the stones by using D-SOC-LL and afterwards balloon and basket catheters [89]. Hence, the potential role of cholangioscopy in the management of hepatolithiasis was underlined. D-SOC-EHL has also been used in hepatolithiasis, with a 57% success rate (four out of seven patients) regarding stone clearance, but a larger sample size is still needed [90]. 

PTC has also been proposed in the treatment of hepatolithiasis, especially in cases of stone distribution to multiple segments, facilitated by laser lithotripsy [91]. 

### 3.6. Stent Placement

In cases where the aforementioned techniques fail and biliary drainage is needed, a temporary plastic stent could be placed as a bridge therapy before proceeding to a second endoscopic stone retrieval attempt or surgical stone removal. According to the latest ESGE guidelines in 2017, a temporary plastic stent should be placed after an unsuccessful ERCP session with a dual role: bile drainage and gradual stone reduction by 50% in 2–6 months [92]. Apart from plastic, ASGE suggests the placement of fully covered self-expandable metal stents too, but with planned stent replacements [5]. It has been supported that mechanical friction between the interface of stones and plastic stents may lead to stone fragmentation [10]. However, stone shrinkage-related guidelines were mainly based on a retrospective study back in 2016 and have not been re-validated prospectively [93], rendering it a topic that warrants further investigation. Another study, published in 2020, explored stents’ potential on stone reduction after an unsuccessful initial ERCP session. It was demonstrated that in a 6-month-period, no stones were found during the second ERCP in 11 out of 46 patients (with an initial median size of 14 mm), while stones were diminished in size in 29 patients (with an initial median size of 19 mm). In six patients with a larger median stone size (26 mm), no difference was found and conventional ERCP methods failed to achieve stone extraction, leading to elective surgery [94]. This study aligned well with the hypothesis of stent usage in terms of stone management. Still, in the era of peroral cholangioscopy techniques, more advanced procedures should be considered. 

## 4. Interventional Radiologist Referral

Frail patients, unfit to undergo surgery, are candidates for PTC and biliary drainage when the previously described endoscopic methods are not feasible [95]. PTC is also suggested in the setting of hepatolithiasis, altered anatomy when the ampulla of Vater is unapproachable, or in secondary sepsis and concomitant hemodynamic instability due to acute cholangitis [49]. During this procedure, a catheter is inserted into the bile ducts under ultrasound guidance followed by fluoroscopy imaging. Balloon dilators as well as mechanical, EHL, and laser lithotripsy can also be used [49,91,96]. Major complications of this technique have an estimated incidence of 2% to 10% and are as follows: infection, bile leak (bilioma), hemorrhage and pneumothorax. Prior to PTC, antibiotic prophylaxis against Gram-negative bacteria is required for all patients. In these cases, PTC, as the last resort, offers valuable treatment for frail patients. 

The combination of PTC and anterograde cholangioscopy is another alternative for the management of bile duct stones, particularly when endoscopic methods are unfeasible. Anterograde cholangioscopy, performed through the percutaneous tract created by PTC, provides a direct visual assessment of the bile ducts, enabling targeted stone fragmentation and extraction. This combination of techniques is especially beneficial in complex cases, offering a minimally invasive approach with a high success rate in stone clearance and the management of related complications.

## 5. Algorithmic Management

**Figure 5:**
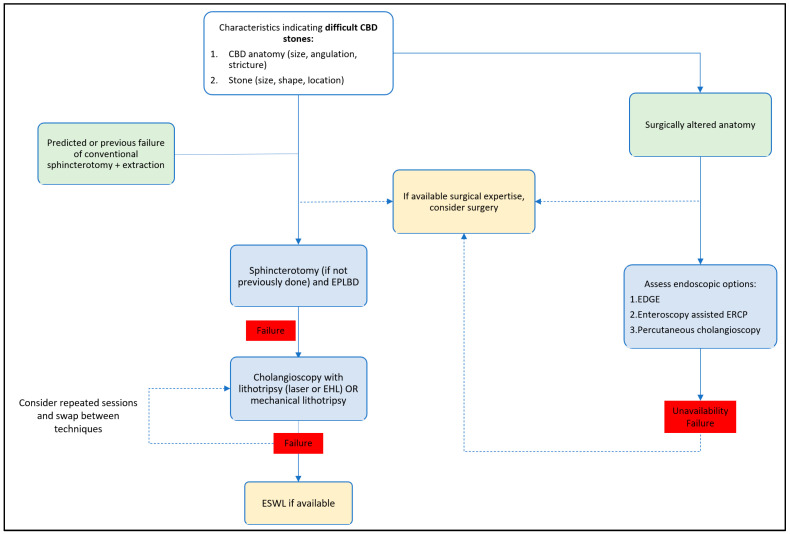
Proposed algorithm for the management of difficult biliary stones. CBD: Common bile duct, EPLBD: endoscopic papillary large balloon dilation, EHL: electrohydraulic lithotripsy, ESWL: extracorporeal shock wave lithotripsy, EDGE: endoscopic ultrasound-directed transgastric endoscopic retrograde cholangiopancreatography, ERCP: endoscopic retrograde cholangiopancreatography.

A proposed algorithm is displayed in Figure 5, about the management of difficult bile duct stones. In the future, a standardized classification scoring system of the variables could stratify the risk–benefit ratio of each technique and propose the ERCP-type (conventional or advanced cholangioscopy). 

## 6. Conclusions

The wide adoption of ERCP techniques across the globe promoted the development of more advanced modalities for stone retrieval during the last decade. Although the majority of stones can be retrieved with a single endoscopic session, 10–15% patients still require different approaches due to either stone-related or biliary duct-related difficulty. Currently, a plethora of instruments is available, so optimum knowledge and use of each accessory is important to provide better clinical outcomes and reduce the cost burden of performing repeated failed endoscopies. 

## Figures and Tables

**Table 1:** Characteristics of difficult biliary stones.

Society	Stone Characteristics					Bile Duct Anatomy	Previous Failed Attempt	Reference
	Size	Number	Position	Shape	Consistency			
ESGE	>1.5	>1	Intrahepatic, Cystic duct, Impaction	Barrel-shaped	N/A	Narrow, short-length or sigmoid-shaped distal CBD/angled CBD	Yes	[3]
ASGE	≥1 cm	>1	Impaction	Eccentric shapes	Hard	Oblique, narrowed, perivaterian distal duct	Yes	[5]
BSG	N/A	N/A	N/A	N/A	N/A	Billroth-II, Roux-en-Y	Yes	[4]

## Data Availability

No data were created during this work.

## Abbreviations

ASGE	American Society for Gastrointestinal Endoscopy
BSG	British Society of Gastroenterology
CBD	common bile duct
DPOC	direct per-oral cholangioscopy
D-SOC	direct per-oral cholangioscopy
EDGE	endoscopic ultrasound-directed transgastric endoscopic retrograde cholangiopancreatography
EHL	electrohydraulic lithotripsy
EPBD	endoscopic papillary balloon dilation
EPLBD	endoscopic papillary large balloon dilation
ERCP	endoscopic retrograde cholangiopancreatography
ESGE	European Society of Gastrointestinal Endoscopy
ESWL	extracorporeal shock wave lithotripsy
EST	endoscopic sphincterotomy
EUS	endoscopic ultrasound
JGES	Japan Gastroenterological Endoscopy Society
LAMS	lumen-apposing metal stent
LCBDE	laparoscopic common bile duct exploration
LL	laser lithotripsy
ML	mechanical lithotripsy
PEP	post-ERPC pancreatitis
PTC	percutaneous transhepatic cholangiography
RCT	randomized control trial
SOC	single-operator cholangioscopy

## References

[1] Tanaja J., Lopez R.A., Meer J.M. (2023). Cholelithiasis. StatPearls [Internet].

[2] McNicoll C.F., Pastorino A., Farooq U., Froehlich M.J., St. Hill S.R. (2023). Choledocholithiasis. StatPearls [Internet].

[3] Manes G., Paspatis G., Aabakken L., Anderloni A., Arvanitakis M., Ah-Soune P., Barthet M., Domagk D., Dumonceau J.-M., Gigot J.-F. (2019). Endoscopic management of common bile duct stones: European Society of Gastrointestinal Endoscopy (ESGE) guideline. Endoscopy.

[4] Williams E., Beckingham I., El Sayed G., Gurusamy K., Sturgess R., Webster G., Young T. (2017). Updated guideline on the management of common bile duct stones (CBDS). Gut.

[5] Buxbaum J.L., Fehmi S.M.A., Sultan S., Fishman D.S., Qumseya B.J., Cortessis V.K., Schilperoort H., Kysh L., Matsuoka L., Yachimski P. (2019). ASGE guideline on the role of endoscopy in the evaluation and management of choledocholithiasis. Gastrointest. Endosc..

[6] Aburajab M., Dua K. (2018). Endoscopic Management of Difficult Bile Duct Stones. Curr. Gastroenterol. Rep..

[7] Troncone E., Mossa M., De Vico P., Monteleone G., Blanco G.D.V. (2022). Difficult Biliary Stones: A Comprehensive Review of New and Old Lithotripsy Techniques. Medicina.

[8] Alexandrino G., Lopes L., Fernandes J., Moreira M., Araújo T., Campos S., Loureiro R., Figueiredo L., Lourenço L.C., Horta D. (2022). Factors Influencing Performance of Cholangioscopy-Guided Lithotripsy Including Available Different Technologies: A Prospective Multicenter Study with 94 Patients. Dig. Dis. Sci..

[9] Brown N.G., Camilo J., Nordstrom E., Yen R.D., Fukami N., Brauer B.C., Wani S., Amateau S.K., Attwell A.R., Shah R.J. (2018). Advanced ERCP techniques for the extraction of complex biliary stones: A single referral center’s 12-year experience. Scand. J. Gastroenterol..

[10] Trikudanathan G., Arain M.A., Attam R., Freeman M.L. (2014). Advances in the endoscopic management of common bile duct stones. Nat. Rev. Gastroenterol. Hepatol..

[11] Subhash A., Buxbaum J.L., Tabibian J.H. (2022). Peroral cholangioscopy: Update on the state-of-the-art. World J. Gastrointest. Endosc..

[12] Sahar N., La Selva D., Gluck M., Gan S.I., Irani S., Larsen M., Ross A.S., Kozarek R.A. (2019). The ASGE grading system for ERCP can predict success and complication rates in a tertiary referral hospital. Surg. Endosc..

[13] Yasuda I., Itoi T. (2013). Recent advances in endoscopic management of difficult bile duct stones. Dig. Endosc..

[14] Veitch A.M., Radaelli F., Alikhan R., Dumonceau J.M., Eaton D., Jerrome J., Lester W., Nylander D., Thoufeeq M., Vanbiervliet G. (2021). Endoscopy in patients on antiplatelet or anticoagulant therapy: British Society of Gastroenterology (BSG) and European Society of Gastrointestinal Endoscopy (ESGE) guideline update. Endoscopy.

[15] Kawai K., Akasaka Y., Murakami K., Tada M., Kohli Y., Nakajima M. (1974). Endoscopic sphincterotomy of the ampulla of Vater. Gastrointest. Endosc..

[16] Köksal A., Eminler A.T., Parlak E. (2018). Biliary endoscopic sphincterotomy: Techniques and complications. World J. Clin. Cases.

[17] Karsenti D., Coron E., Vanbiervliet G., Privat J., Kull E., Bichard P., Perrot B., Quentin V., Duriez A., Cholet F. (2017). Complete endoscopic sphincterotomy with vs. without large-balloon dilation for the removal of large bile duct stones: Randomized multicenter study. Endoscopy.

[18] Stefanidis G., Christodoulou C., Manolakopoulos S., Chuttani R. (2012). Endoscopic extraction of large common bile duct stones: A review article. World J. Gastrointest. Endosc..

[19] Jeong S.U. (2013). Endoscopic papillary balloon dilation: Revival of the old technique. World J. Gastroenterol..

[20] Testoni P.A., Mariani A., Aabakken L., Arvanitakis M., Bories E., Costamagna G., Devière J., Dinis-Ribeiro M., Dumonceau J.-M., Giovannini M. (2016). Papillary cannulation and sphincterotomy techniques at ERCP: European Society of Gastrointestinal Endoscopy (ESGE) Clinical Guideline. Endoscopy.

[21] Kuo Y.-T., Wang H.-P., Chang C.-Y., Leung J.W., Chen J.-H., Tsai M.-C., Liao W.-C. (2017). Comparable Long-term Outcomes of 1-Minute vs. 5-Minute Endoscopic Papillary Balloon Dilation for Bile Duct Stones. Clin. Gastroenterol. Hepatol..

[22] Chou C.-K., Lee K.-C., Luo J.-C., Chen T.-S., Perng C.-L., Huang Y.-H., Lin H.-C., Hou M.-C. (2020). Endoscopic papillary balloon dilatation less than three minutes for biliary stone removal increases the risk of post-ERCP pancreatitis. PLoS ONE.

[23] Lee D., Lee B., Niwa H., Tajiri H., Nakajima M., Yasuda K. (2008). EST, EPBD, and EPLBD (Cut, Stretch, or Both?). New Challenges in Gastrointestinal Endoscopy.

[24] Baron T., Harewood G. (2004). Endoscopic Balloon Dilation of the Biliary Sphincter Compared to Endoscopic Biliary Sphincterotomy for Removal of Common Bile Duct Stones During ERCP: A Meta-analysis of Randomized, Controlled Trials. Am. J. Gastroenterol..

[25] Liu Y., Su P., Lin S., Xiao K., Chen P., An S., Zhi F., Bai Y. (2012). Endoscopic papillary balloon dilatation versus endoscopic sphincterotomy in the treatment for choledocholithiasis: A meta-analysis. J. Gastroenterol. Hepatol..

[26] Weinberg B., Shindy W., Lo S. (2006). Endoscopic balloon sphincter dilation (sphincteroplasty) versus sphincterotomy for common bile duct stones. Emergencias.

[27] Dong S.Q., Singh T.P., Zhao Q., Li J.J., Wang H.L. (2019). Sphincterotomy plus balloon dilation versus sphincterotomy alone for choledocholithiasis: A meta-analysis. Endoscopy.

[28] Itoi T., Ryozawa S., Katanuma A., Okabe Y., Kato H., Horaguchi J., Tsuchiya T., Gotoda T., Fujita N., Yasuda K. (2018). Japan Gastroenterological Endoscopy Society guidelines for endoscopic papillary large balloon dilation. Dig. Endosc..

[29] Fujisawa T., Kagawa K., Hisatomi K., Kubota K., Nakajima A., Matsuhashi N. (2016). Is endoscopic papillary balloon dilatation really a risk factor for post-ERCP pancreatitis?. World J. Gastroenterol..

[30] Kim J.H. (2013). Endoscopic papillary large balloon dilation for the removal of bile duct stones. World J. Gastroenterol..

[31] Chiu Y.-C., Liang C.-M., Wu C.-K., Lu L.-S., Tai W.-C., Kuo Y.-H., Wu K.-L., Chuah S.-K., Kuo C.-H. (2019). The efficacy of limited endoscopic sphincterotomy plus endoscopic papillary large balloon dilation for removal of large bile duct stones. BMC Gastroenterol..

[32] Kogure H., Kawahata S., Mukai T., Doi S., Iwashita T., Ban T., Ito Y., Kawakami H., Hayashi T., Sasahira N. (2020). Multicenter randomized trial of endoscopic papillary large balloon dilation without sphincterotomy versus endoscopic sphincterotomy for removal of bile duct stones: MARVELOUS trial. Endoscopy.

[33] Park J.-S., Jeong S., Lee D.K., Jang S.I., Lee T.H., Park S.-H., Hwang J.C., Kim J.H., Yoo B.M., Park S.G. (2018). Comparison of endoscopic papillary large balloon dilation with or without endoscopic sphincterotomy for the treatment of large bile duct stones. Endoscopy.

[34] Guo Y., Lei S., Gong W., Gu H., Li M., Liu S., Zhi F. (2015). A Preliminary Comparison of Endoscopic Sphincterotomy, Endoscopic Papillary Large Balloon Dilation, and Combination of the Two in Endoscopic Choledocholithiasis Treatment. J. Pharmacol. Exp. Ther..

[35] Liu P., Lin H., Chen Y., Wu Y.-S., Tang M., Lai L. (2019). Comparison of endoscopic papillary large balloon dilation with and without a prior endoscopic sphincterotomy for the treatment of patients with large and/or multiple common bile duct stones: A systematic review and meta-analysis. Ther. Clin. Risk Manag..

[36] Ryozawa S., Itoi T., Katanuma A., Okabe Y., Kato H., Horaguchi J., Fujita N., Yasuda K., Tsuyuguchi T., Fujimoto K. (2018). Japan Gastroenterological Endoscopy Society guidelines for endoscopic sphincterotomy. Dig. Endosc..

[37] Park C.H., Jung J.H., Nam E., Kim E.H., Kim M.G., Kim J.H., Park S.W. (2018). Comparative efficacy of various endoscopic techniques for the treatment of common bile duct stones: A network meta-analysis. Gastrointest. Endosc..

[38] Meng W., Leung J.W., Zhang K., Zhou W., Wang Z., Zhang L., Sun H., Xue P., Liu W., Wang Q. (2019). Optimal dilation time for combined small endoscopic sphincterotomy and balloon dilation for common bile duct stones: A multicentre, single-blinded, randomised controlled trial. Lancet Gastroenterol. Hepatol..

[39] Youn Y.H., Lim H.C., Jahng J.H., Jang S.I., You J.H., Park J.S., Lee S.J., Lee D.K. (2011). The Increase in Balloon Size to Over 15 mm Does Not Affect the Development of Pancreatitis After Endoscopic Papillary Large Balloon Dilatation for Bile Duct Stone Removal. Dig. Dis. Sci..

[40] Omnimed (2023). Retrieval Baskets & Nets. https://www.omnimed.co.uk/products/retrieval-baskets-nets.

[41] Saito H., Iwasaki H., Itoshima H., Kadono Y., Shono T., Kamikawa K., Urata A., Nasu J., Uehara M., Matsushita I. (2023). Comparison of Outcomes between a Basket Catheter and a Balloon Catheter for Endoscopic Common Bile Duct Stone Removal. Dig. Dis..

[42] Sharma R., Sharma V., Singhal U., Sanaka M. (2022). Outcomes of balloon vs basket catheter for clearance of choledocholithiasis: A systematic review and meta-analysis. Endosc. Int. Open.

[43] Inoue T., Ibusuki M., Kitano R., Sakamoto K., Kimoto S., Kobayashi Y., Sumida Y., Nakade Y., Ito K., Yoneda M. (2023). Comparison of the mechanical properties of retrieval basket catheters for bile duct stones: An experimental study. Indian J. Gastroenterol..

[44] Tringali A., Costa D., Fugazza A., Colombo M., Khalaf K., Repici A., Anderloni A. (2021). Endoscopic management of difficult common bile duct stones: Where are we now? A comprehensive review. World J. Gastroenterol..

[45] Watson R.R., Parsi M.A., Aslanian H.R., Goodman A.J., Lichtenstein D.R., Melson J., Navaneethan U., Pannala R., Sethi A., Sullivan S.A. (2018). Biliary and pancreatic lithotripsy devices. VideoGIE.

[46] Chang W.-H., Chu C.-H., Wang T.-E., Chen M.-J., Lin C.-C. (2004). Outcome of simple use of mechanical lithotripsy of difficult common bile duct stones. World J. Gastroenterol..

[47] Kamezaki H., Iwanaga T., Tokunaga M., Maeda T., Senoo J.-I., Ohyama H., Kato N. (2023). Addition of Mechanical Lithotripsy to Endoscopic Papillary Large Balloon Dilation in Patients with Difficult Common Bile Duct Stones: A Retrospective Single-Center Study. J. Laparoendosc. Adv. Surg. Tech..

[48] Radwan M.I., Emara M.H., Ibrahim I.M., Moursy M.E. (2019). Large Balloon Dilatation Versus Mechanical Lithotripsy After Endoscopic Sphincterotomy in the Management of Large Common Bile Duct Stones in Cirrhotic Patients: A Randomized Study. J. Clin. Gastroenterol..

[49] Narula V.K., Fung E.C., Overby D.W., Richardson W., Stefanidis D., SAGES Guidelines Committee Clinical Spotlight Review: Management of Choledocholithiasis. https://www.sages.org/publications/guidelines/clinical-spotlight-review-management-of-choledocholithiasis/.

[50] Tao T., Zhang M., Zhang Q.-J., Li L., Li T., Zhu X., Li M.-D., Li G.-H., Sun S.-X. (2017). Outcome of a session of extracorporeal shock wave lithotripsy before endoscopic retrograde cholangiopancreatography for problematic and large common bile duct stones. World J. Gastroenterol..

[51] Parsa N., Khashab M.A. (2019). The Role of Peroral Cholangioscopy in Evaluating Indeterminate Biliary Strictures. Clin. Endosc..

[52] Draganov P. (2008). The SpyGlass^®^ Direct Visualization System for Cholangioscopy. Gastroenterol. Hepatol..

[53] Mizrahi M., Khoury T., Wang Y., Cohen J., Sheridan J., Chuttani R., Berzin T.M., Sawhney M.S., Pleskow D.K. (2018). “Apple Far from the Tree”: Comparative effectiveness of fiberoptic single-operator cholangiopancreatoscopy (FSOCP) and digital SOCP (DSOCP). HPB.

[54] Thaker A.M., Muthusamy V.R. (2017). The role and utility of cholangioscopy for diagnosing indeterminate biliary strictures. Int. J. Gastrointest. Interv..

[55] Yang J.-J., Liu X.-C., Chen X.-Q., Zhang Q.-Y., Liu T.-R. (2019). Clinical value of DPOC for detecting and removing residual common bile duct stones (video). BMC Gastroenterol..

[56] Sliney D.H., Trokel S.L. (1993). Medical Lasers and Their Safe Use.

[57] Patel S.N., Rosenkranz L., Hooks B., Tarnasky P.R., Raijman I., Fishman D.S., Sauer B.G., Kahaleh M. (2014). Holmium-yttrium aluminum garnet laser lithotripsy in the treatment of biliary calculi using single-operator cholangioscopy: A multicenter experience (with video). Gastrointest. Endosc..

[58] Koch H., Rösch W., Walz V. (1980). Endoscopic lithotripsy in the common bile duct. Gastrointest. Endosc..

[59] Vorreuther R., Engelmann Y. (1995). Evaluation of the shock-wave pattern for endoscopic electrohydraulic lithotripsy. Surg. Endosc..

[60] Kamiyama R., Ogura T., Okuda A., Miyano A., Nishioka N., Imanishi M., Takagi W., Higuchi K. (2018). Electrohydraulic Lithotripsy for Difficult Bile Duct Stones under Endoscopic Retrograde Cholangiopancreatography and Peroral Transluminal Cholangioscopy Guidance. Gut Liver.

[61] Buxbaum J., Sahakian A., Ko C., Jayaram P., Lane C., Yu C.Y., Kankotia R., Laine L. (2018). Randomized trial of cholangioscopy-guided laser lithotripsy versus conventional therapy for large bile duct stones (with videos). Gastrointest. Endosc..

[62] Bang J.Y., Sutton B., Navaneethan U., Hawes R., Varadarajulu S. (2020). Efficacy of Single-Operator Cholangioscopy-Guided Lithotripsy Compared with Large Balloon Sphincteroplasty in Management of Difficult Bile Duct Stones in a Randomized Trial. Clin. Gastroenterol. Hepatol..

[63] Angsuwatcharakon P., Kulpatcharapong S., Ridtitid W., Boonmee C., Piyachaturawat P., Kongkam P., Pareesri W., Rerknimitr R. (2019). Digital cholangioscopy-guided laser versus mechanical lithotripsy for large bile duct stone removal after failed papillary large-balloon dilation: A randomized study. Endoscopy.

[64] Turowski F., Hügle U., Dormann A., Bechtler M., Jakobs R., Gottschalk U., Nötzel E., Hartmann D., Lorenz A., Kolligs F. (2018). Diagnostic and therapeutic single-operator cholangiopancreatoscopy with SpyGlassDSTM: Results of a multicenter retrospective cohort study. Surg. Endosc..

[65] Fugazza A., Gabbiadini R., Tringali A., De Angelis C.G., Mosca P., Maurano A., Di Mitri R., Manno M., Mariani A., Cereatti F. (2022). Digital single-operator cholangioscopy in diagnostic and therapeutic bilio-pancreatic diseases: A prospective, multicenter study. Dig. Liver Dis..

[66] McCarty T.R., Gulati R., Rustagi T. (2021). Efficacy and safety of peroral cholangioscopy with intraductal lithotripsy for difficult biliary stones: A systematic review and meta-analysis. Endoscopy.

[67] Gutierrez O.I.B., Bekkali N.L., Raijman I., Sturgess R., Sejpal D.V., Aridi H.D., Sherman S., Shah R.J., Kwon R.S., Buxbaum J.L. (2018). Efficacy and Safety of Digital Single-Operator Cholangioscopy for Difficult Biliary Stones. Clin. Gastroenterol. Hepatol..

[68] Facciorusso A., Gkolfakis P., Ramai D., Tziatzios G., Lester J., Crinò S.F., Frazzoni L., Papanikolaou I.S., Arvanitakis M., Blero D. (2021). Endoscopic Treatment of Large Bile Duct Stones: A Systematic Review and Network Meta-Analysis. Clin. Gastroenterol. Hepatol..

[69] Huang L., Xu Y., Chen J., Liu F., Wu D., Zhou W., Wu L., Pang T., Huang X., Zhang K. (2023). An artificial intelligence difficulty scoring system for stone removal during ERCP: A prospective validation. Endoscopy.

[70] Teoh A.Y.B., Cheung F.K.Y., Hu B., Pan Y.M., Lai L.H., Chiu P.W.Y., Wong S.K.H., Chan F.K.L., Lau J.Y.W. (2013). Randomized Trial of Endoscopic Sphincterotomy with Balloon Dilation Versus Endoscopic Sphincterotomy Alone for Removal of Bile Duct Stones. Gastroenterology.

[71] Paik W.H., Ryu J.K., Park J.M., Song B.J., Kim J., Park J.K., Kim Y.-T. (2014). Which Is the Better Treatment for the Removal of Large Biliary Stones? Endoscopic Papillary Large Balloon Dilation versus Endoscopic Sphincterotomy. Gut Liver.

[72] Conigliaro R., Camellini L., Zuliani C.G., Sassatelli R., Mortilla M.G., Bertoni G., Formisano D., Bedogni G. (2006). Clearance of Irretrievable Bile Duct and Pancreatic Duct Stones by Extracorporeal Shockwave Lithotripsy, Using a Transportable Device: Effectiveness and Medium-term Results. J. Clin. Gastroenterol..

[73] Alrajhi S., Barkun A., Adam V., Callichurn K., Martel M., Brewer O., Khashab M.A., Forbes N., Almadi M.A., Chen Y.I. (2021). Early cholangioscopy-assisted electrohydraulic lithotripsy in difficult biliary stones is cost-effective. Ther. Adv. Gastroenterol..

[74] Deprez P.H., Duran R.G., Moreels T., Furneri G., Demma F., Verbeke L., Van der Merwe S.W., Laleman W. (2018). The economic impact of using single-operator cholangioscopy for the treatment of difficult bile duct stones and diagnosis of indeterminate bile duct strictures. Endoscopy.

[75] Sljivic I., Trasolini R., Donnellan F. (2022). Cost-effective analysis of preliminary single-operator cholangioscopy for management of difficult biliary stones. Endosc. Int. Open.

[76] Dumonceau J.-M., Andriulli A., Elmunzer B.J., Mariani A., Meister T., Deviere J., Marek T., Baron T.H., Hassan C., Testoni P.A. (2014). Prophylaxis of post-ERCP pancreatitis: European Society of Gastrointestinal Endoscopy (ESGE) Guideline—Updated June 2014. Endoscopy.

[77] Bokun T., Tadic M., Kurtcehajic A., Grgurevic I., Kujundzic M. (2020). Broken handle cord of impacted biliary basket—Rescue by cholangioscopy with laser lithotripsy. Endoscopy.

[78] Libânio D., Giestas S., Martinez-Ares D., Canena J., Lopes L. (2018). Cholangioscopy-guided holmium laser lithotripsy of a stone trapped in a mechanical lithotripter. VideoGIE.

[79] Oh C.H., Dong S.H. (2021). Recent advances in the management of difficult bile-duct stones: A focus on single-operator cholangioscopy-guided lithotripsy. Korean J. Intern. Med..

[80] Korrapati P., Ciolino J., Wani S., Shah J., Watson R., Muthusamy V.R., Klapman J., Komanduri S. (2016). The efficacy of peroral cholangioscopy for difficult bile duct stones and indeterminate strictures: A systematic review and meta-analysis. Endosc. Int. Open.

[81] Galetti F., de Moura D.T.H., Ribeiro I.B., Funari M.P., Coronel M., Sachde A.H., Brunaldi V.O., Franzini T.P., Bernardo W.M., de Moura E.G.H. (2020). Cholangioscopy-guided lithotripsy vs. conventional therapy for complex bile duct stones: A systematic review and meta-analysis. ABCD Arq. Bras. Cir. Dig..

[82] Ali M.F., Modayil R., Gurram K.C., Brathwaite C.E., Friedel D., Stavropoulos S.N. (2018). Spiral enteroscopy-assisted ERCP in bariatric-length Roux-en-Y anatomy: A large single-center series and review of the literature (with video). Gastrointest. Endosc..

[83] Espinel Díez J., Pinedo Ramos M.E. (2020). Single-balloon enteroscopy-assisted ERCP in patients with Roux-en-Y anatomy and choledocholithiasis: Do technical improvements mean better outcomes?. Rev. Esp. Enferm. Dig..

[84] Kedia P., Sharaiha R.Z., Kumta N.A., Kahaleh M. (2014). Internal EUS-Directed Transgastric ERCP (EDGE): Game Over. Gastroenterology.

[85] James T.W., Baron T.H. (2019). Endoscopic Ultrasound-Directed Transgastric ERCP (EDGE): A Single-Center US Experience with Follow-up Data on Fistula Closure. Obes. Surg..

[86] Dhindsa B.S., Dhaliwal A., Mohan B.P., Mashiana H.S., Girotra M., Singh S., Ohning G., Bhat I., Adler D.G. (2020). EDGE in Roux-en-Y gastric bypass: How does it compare to laparoscopy-assisted and balloon enteroscopy ERCP: A systematic review and meta-analysis. Endosc. Int. Open.

[87] Sato T., Nakai Y., Kogure H., Mitsuyama T., Shimatani M., Uemura S., Iwashita T., Tanisaka Y., Ryozawa S., Tsuchiya T. (2024). ERCP using balloon-assisted endoscopes versus EUS-guided treatment for common bile duct stones in Roux-en-Y gastrectomy. Gastrointest. Endosc..

[88] Martínez-Moreno B., Casellas J.A., Tormo J.R.A. (2020). Endoscopic ultrasound-guided gastrojejunostomy-assisted ERCP in a Billroth II gastrectomy patient. Endoscopy.

[89] Liu R., Zhang B., Liu D. (2018). Peroral cholangioscopy-guided laser lithotripsy to treat regional hepatolithiasis without stricture. Dig. Endosc..

[90] Mansilla-Vivar R., Alonso-Lázaro N., Argüello-Viudez L., Ponce-Romero M., Bustamante-Balen M., Pons-Beltrán V. (2020). New management of hepatolithiasis: Can surgery be avoided? (with video). Gastroenterol. Hepatol..

[91] Lamanna A., Maingard J., Tai J., Ranatunga D., Goodwin M. (2019). Percutaneous transhepatic Laser lithotripsy for intrahepatic cholelithiasis. Diagn. Interv. Imaging.

[92] Dumonceau J.M., Tringali A., Blero D., Devière J., Laugiers R., Heresbach D., Costamagna G. (2018). Endoscopic biliary stenting: Indications, choice of stents, and results: European Society of Gastrointestinal Endoscopy (ESGE) Clinical Guideline—Updated October 2017. Endoscopy.

[93] Mohammed N., Pinder M., Harris K., Everett S.M. (2016). Endoscopic biliary stenting in irretrievable common bile duct stones: Stent exchange or expectant management—Tertiary-centre experience and systematic review. Front. Gastroenterol..

[94] Omar A.N. (2020). Role of biliary stenting for large impacted stone in common bile duct. Egypt. J. Surg..

[95] Young M., Mehta D. (2023). Percutaneous Transhepatic Cholangiogram. StatPearls [Internet].

[96] Ierardi A.M., Rodà G.M., Di Meglio L., Pellegrino G., Cantù P., Dondossola D., Rossi G., Carrafiello G. (2021). Percutaneous Transhepatic Electrohydraulic Lithotripsy for the Treatment of Difficult Bile Stones. J. Clin. Med..

